# Urinary Trypsin Inhibitor Ameliorates Seawater Immersion-Induced Intestinal Mucosa Injury via Antioxidation, Modulation of NF-**κ**B Activity, and Its Related Cytokines in Rats with Open Abdominal Injury

**DOI:** 10.1155/2014/858237

**Published:** 2014-08-21

**Authors:** Xing Jian Zhang, Ya Li Wang, Song Zhou, Xiaojun Xue, Qiang Liu, Wen Hua Zhang, Jun Zheng

**Affiliations:** ^1^Department of General Surgery, The 175th Hospital of PLA, Southeast Hospital Affiliated to Xiamen University, No. 269 Zhanghua Middle Road, Zhangzhou, Fujian 363000, China; ^2^The First College of Clinical Medical Science, China Three Gorges University, No. 183 Yiling Road, Yichang, Hubei 443000, China

## Abstract

*Objective*. To investigate the role of oxidative stress, NF-*κ*B activity, and its related cytokines in the pathogenesis of seawater immersion after open abdominal injury (SI-OAI) and whether UTI treatment can attenuate SI-OAI induced IMI.* Methods*. Wistar rats were randomly divided into three groups: C group, S group, and U group. The rats in C group only suffered from anesthesia and surgical operation, whereas the rats in S group and U group received caudal vein injection of normal saline without/with 50,000 U/kg body weight of UTI. The activities of TNF-*α*, IL-6, SOD, MDA, ROS, NF-*κ*B, and I*κ*B-*β* were monitored by ELISA, biochemical methods, EMSA, and Western blot, respectively.* Results*. The plasma inflammatory mediators and the contents of MDA, ROS, and NF-*κ*B in intestine as well as the pathological scores in ileal mucosa were significantly increased in rats after SI-OAI, accompanied by a reduction in SOD activities and I*κ*B-*β* levels. UTI treatment significantly attenuated intestinal histopathological changes with evidence of a decrease in all of the parameters, except for upregulation of the levels of SOD and I*κ*B-*β* protein.* Conclusion*. UTI can attenuate SI-OAI induced IMI via inhibition of NF-*κ*B activity, subsequently inhibiting the expression of inflammatory cytokines and by combating oxidative stress.

## 1. Introduction

Increasing evidence suggests that the intestinal mucosa injury (IMI) plays pivotal roles in the pathological progression of the inflammatory and metabolic responses to sepsis, trauma, and other critical illnesses [1, 2]. Major trauma and sepsis may undermine the intestinal mucosa, initiating a cascade of intestinal events such as increased intestinal permeability, translocation of intestinal bacteria, and endotoxemia, followed by remote organs and tissues damage, finally leading to systemic inflammatory response syndrome (SIRS) and multiple organ dysfunction syndrome (MODS) [3, 4]. Previous studies in Chinese literature showed that SI-OAI could induce marked damage of intestinal mucosa structure in animal models [5–7]. The pathogenesis of SI-IMI still remains obscure and multifactorial. NF-*κ*B is a common transcription factor which plays an important role in the pathogenesis of IMI induced by SI-OAI. In SI-OAI rat model, the activation of NF-*κ*B, followed by the excessive release of cytokines, such as tumor necrosis factor-alpha (TNF-*α*) and interleukin-1 (IL-1) in intestinal tissue, played an important role in acute gut mucosal injury [8, 9]. Besides, participation of reactive oxygen species (ROS) acted as an essential trigger in the pathogenesis of SI-OAI induced IMI [10]. Therefore, inhibition of inflammation and oxidative stress during the early phase of SI-OAI may arrest the pathologic progression that leads to significant morbidity and mortality.

Urinary trypsin inhibitor (UTI), a serine-type protease inhibitor purified from the fresh urine of healthy men, decreases the protease secretion from neutrophils and suppresses the activity of neutrophil elastase. It has been widely used in the treatment of circulatory shock, septic shock, and pancreatitis [11–13]. Several studies in vitro have demonstrated that UTI may protect against systemic inflammation by suppressing the activation of NF-*κ*B and the local expression of proinflammatory cytokines [14, 15]. Furthermore, few other studies described UTI as a compound possessing antioxidant properties in endotoxemic rats [16, 17]. These findings suggest that UTI can ameliorate organ injury in severe injuries by suppressing systemic inflammation and oxidative stress.

To our best knowledge, no study was found in the literature to investigate whether UTI has protective effects in the development of IMI after SI-OAI. Therefore, this study was aimed to evaluate the potential protective effects of UTI in SI-OAI by inhibiting activation of NF-*κ*B, excess expression of cytokines, and oxidative stress in a rat model.

## 2. Materials and Methods

### 2.1. Ethics Statement

This study was approved by the Animal Experiment Committee of Xiamen University and all the rats were handled according to the Declaration of the National Institutes of Health Guide for Care and Use of Laboratory Animals.

### 2.2. Drug and Reagents

UTI was obtained from Pharmacy Department of the Southeast Hospital affiliated to Xiamen University. Seawater was prepared according to the standard of the Third Institute of Oceanography of the State Ocean Bureau [18]. Enzyme-linked immunosorbent assay (ELISA) kits of TNF-*α* and IL-6 as well as malondialdehyde (MDA) and superoxide dismutase (SOD) assay kits were purchased from Jiancheng Bioengineering Institute (Nanjing, Jiangsu, China). Nonradioactive electrophoretic mobility shift assay (EMSA) kit, bicinchoninic acid (BCA) protein assay reagent kit, and reactive oxygen species assay kit were purchased from Beyotime Institute of Biotechnology (Heimen, Jiangsu, China). The I*κ*B-*β* antibody and anti-*β*-actin monoclonal antibodies were purchased from Santa Cruz Biotechnology Inc. (Santa Cruz, CA, USA).

### 2.3. Animal Preparation

Male Wistar rats (240–280 g) were obtained from the animal center of Xiamen University. All animals were kept under specific pathogen-free conditions with controlled light/dark cycles and free access to water and standard chow and were fasted for 24 hours before surgical procedures. All experiments were started at 8 am to wipe out the influences of biorhythm on experimental results. 40 male Wistar rats were randomly assigned to 3 groups, control group (C group), seawater immersion group (S group), and UTI treatment group (U group). S and U groups included 2 time subgroups, 3 and 6 h. There were 8 rats in each subgroup.

### 2.4. Experimental Protocols

The SI-OAI model was produced based on the method described by Han et al. [8]. Briefly, the rats were anesthetized with xylazine hydrochloride injection (1 mL/kg of body wt, administered i.m.) and then fixed with supine position on a self-made plate; a ventral midline incision of 3 cm was performed by eye scissors to create a surgical abdominal open injury. The immersion surface was evened with the xiphoid. Rats were pulled up after immersion in 22°C seawater for 1 h and then the abdominal injuries were sutured immediately. Disinfected cotton ball was used to absorb the seawater in the abdominal cavity. In the S group, the rats received tail vein injection of 3 mL normal saline (NS) immediately after the immersion of seawater; in the U group, the rats received 3 mL UTI (50,000 U/kg) immediately in the same way; in the C group, the rats received the same anesthesia and surgical operation as described, but neither seawater immersion nor UTI or NS treatment was performed.

### 2.5. Sample Processing

The rats were sacrificed to acquire the blood and intestinal tissues. Blood samples in the S and U groups were taken by cardiac puncture for the measurement of TNF-*α* and IL-6 at 3 and 6 h after immersion of seawater. Blood samples in the C group were collected in the same way at 1 h after the experiment. Then they were collected in tubes containing EDTA at baseline and separated by centrifugation; the plasma was stored at −20°C until use. The small intestine from the Treitz ligament to the ileocecal junction was rapidly excised from the mesentery and was rinsed gently by 5 mL ice-cold PBS. A 3 cm segment of ileum at the distance of 15 cm upward away from ileocecal junction was removed for histopathological studies. The others were stored in liquid nitrogen immediately until tissue assays.

### 2.6. Determination of TNF-*α* and IL-6 Expression Levels

TNF-*α* and IL-6 levels were determined in plasma samples using the commercially available enzyme-linked immunosorbent assay (ELISA) kits. The measurement was performed step by step based on the protocol booklet. Samples were reacted with the assay reagents in the ELISA kit, respectively, and were analyzed spectrophotometrically at 450 nm of absorbance. The results were expressed as pg/mL.

### 2.7. Determination of Biochemical Parameters

The frozen small intestine tissue (about 1 cm) was fixed with 3-fold phosphate buffer (0.1 M, pH 7.2), homogenated, and centrifugalized (10000 rpm/min, 30 min) for biochemical parameters estimation. The intestinal lipid peroxidation level was determined by estimating MDA in the tissue supernatants with a colorimetric assay kit specific for MDA. SOD levels in the tissue supernatants were measured using the colorimetric method according to the manufacturer's protocol. The production of free radicals (ROS) in the tissue supernatants was determined by using 2′,7′-dichlorofluorescin diacetate (DCFH-DA) staining as described previously [19]. The fluorescence was measured by spectrofluorometer with an excitation at 488 nm and emission at 525 nm.

### 2.8. Electrophoretic Mobility Shift Assay (EMSA)

Activation of NF-*κ*B was examined using a commercial nonradioactive EMSA kit according to the manufacturer's protocol. Nuclear protein prepared from intestinal tissue was extracted and quantified as described [20]. The double-stranded 5′-biotin-labeled NF-*κ*B oligonucleotide probe (5′-AGT TGA GGG GAC TTT CCC AGGC-3′) was incubated with the nuclear extract in a binding buffer for 30 min at room temperature. After the reaction the DNA-protein complexes were subjected to a 6% native polyacrylamide DNA retardation gel and electroblotted onto positively charged nylon membranes. Then, the membrane was immediately cross-linked for 10 minutes with a UV cross-linker, followed by treatment with a 1 : 750 diluted streptavidin-HRP conjugate. Then, the chemiluminence substrate buffer was added and resulting chemiluminescence was detected by autoradiography.

### 2.9. Western Blot

Western blotting was performed for analysis of I*κ*B-*β* protein formation according to the traditional protocol. Protein contents in frozen intestinal tissue samples were determined using a BCA protein assay reagent kit. The proteins were separated with 12% sodium dodecyl sulfate-polyacrylamide (SDS-PAGE) gel electrophoresis and transferred to nitrocellulose. The membranes were blocked with 3% bovine serum albumin (BSA) for 2 h and thoroughly washed with PBST (phosphate-buffered saline with 0.1% Tween 80) and were probed with primary antibodies (I*κ*B-*β* antibody) in 1 : 800 dilution overnight at 4°C. After washing with PBST, the membranes were incubated with the secondary antibodies, goat anti-mouse IgG/HRP in 1 : 2000 dilution for 1 h at room temperature. Then, the membranes were washed with PBST 4 times (10 min for each). Protein bands were located with enhanced chemiluminescence (ECL) system. The optical density of bands was measured with computer-assisted densitometric analysis of the exposed films.

### 2.10. Histopathological Examination

The paraformaldehyde-fixed ileum was embedded in paraffin, sectioned at 4 *μ*m thickness with a microtome, and stained with hematoxylin and eosin (H&E) for histological examination. Damage of intestinal mucosa was examined by two pathologists who were blinded to the study groups. The damage of the specimens was assessed semiquantitatively according to changes of the villus and glands of the intestinal mucosa described by Chiu et al. [21]: grade 0, normal mucosal villi; grade 1, development of subepithelial space; grade 2, extension of the subepithelial space with moderate lifting of the epithelial layer from the lamina propria; grade 3, massive epithelial lifting down the sides of villi, possibly with a few denuded tips; grade 4, denuded villi with lamina propria and dilated capillaries exposed; and grade 5, digestion and disintegration of the lamina propria, hemorrhage, and ulceration. A minimum of six randomly chosen fields from each rat were evaluated and averaged to determine mucosal damage.

### 2.11. Statistical Methods

Statistics were analyzed with SPSS 17.0 software (SPSS Inc., Chicago, IL, USA). All data are expressed as mean ± SD. One-way repeated measures analysis of variance (ANOVA) was used for multiple comparisons. The Chi-square test was used to compare categorical variables. *P* < 0.05 was considered statistically significant.

## 3. Results

### 3.1. Effects of UTI Treatment on Plasma Levels of TNF-*α* and IL-6

**Figure 1:**
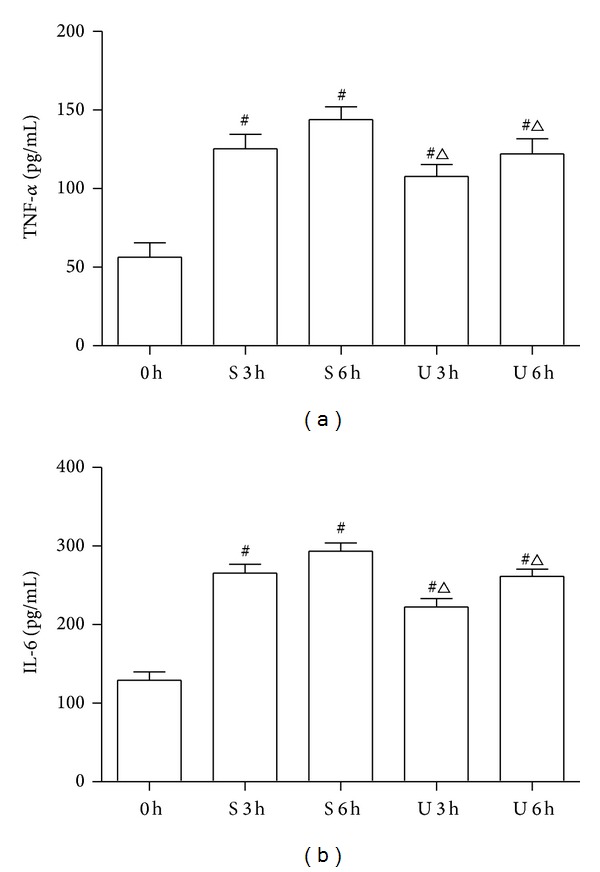
Effects of UTI on TNF-*α* (a) and IL-6 (b). 0 h = C group; S 3 h = S group at 3 h; S 6 h = S group at 6 h; U 3 h = U group at 3 h; U 6 h = U group at 6 h. ^#^ *P* < 0.05 versus C group and ^△^ *P* < 0.05 versus S group at matched time.

Concentrations of TNF-*α* and IL-6 were significantly upregulated in rats of S and U groups compared with those of C group at all time points (*P* < 0.01). UTI markedly inhibited the expression of these cytokines in the rats with SI-OAI compared with those of S group (*P* < 0.05) (Figure 1).

### 3.2. Effects of UTI Treatment on ROS in the Tissue Supernatants

**Figure 2:**
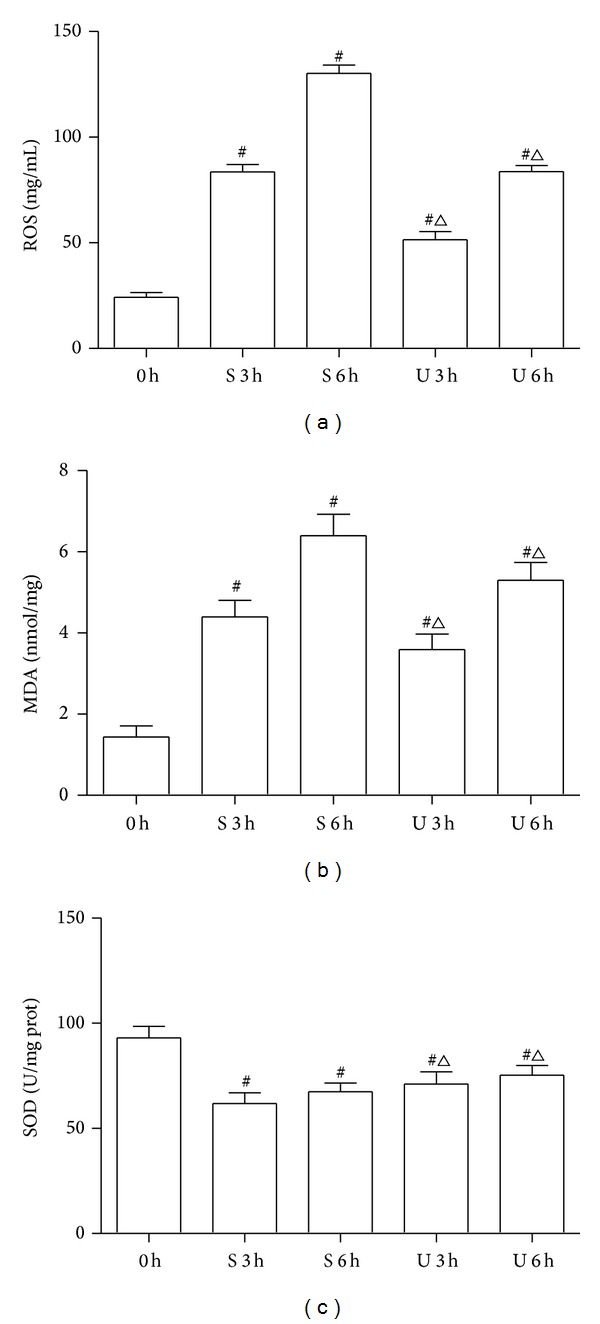
Effects of UTI on ROS (a), MDA (b), and SOD (c) of intestinal tissue. 0 h = C group; S 3 h = S group at 3 h; S 6 h = S group at 6 h; U 3 h = U group at 3 h; U 6 h = U group at 6 h. ^#^ *P* < 0.05 versus C group and ^△^ *P* < 0.05 versus S group at matched time.

The level of 2′-7′-dichlorofluorescein (DCFH) in the tissue supernatants was determined as an index of the ROS production. As shown in Figure 2(a), SI-OAI caused a significant increase in DCFH oxidation in S and U groups compared with C group (*P* < 0.05). The expression levels of DCFH were higher in U group than in C group but lower than in S group (*P* < 0.05).

### 3.3. Effects of UTI Treatment on MDA and SOD in the Tissue Supernatants

MDA levels in the tissue supernatants were detected to assess lipid peroxidation. As shown in Figure 2(b), MDA levels were significantly increased in S and U groups in comparison to those in C group (*P* < 0.01). UTI treatment obviously prevented the increased MDA activity compared with those in S group (*P* < 0.01). On the contrary, SI-OAI caused a statistically significant decrease in SOD levels in S and U groups compared with those in C group (*P* < 0.05). UTI treatment obviously prevented the decreased SOD activity induced by SI-OAI compared with S group (*P* < 0.05, Figure 2(c)).

### 3.4. Effects of UTI Treatment on NF-*κ*B Activity and I*κ*B-*β* Expression in Intestinal Homogenate

**Figure 3:**
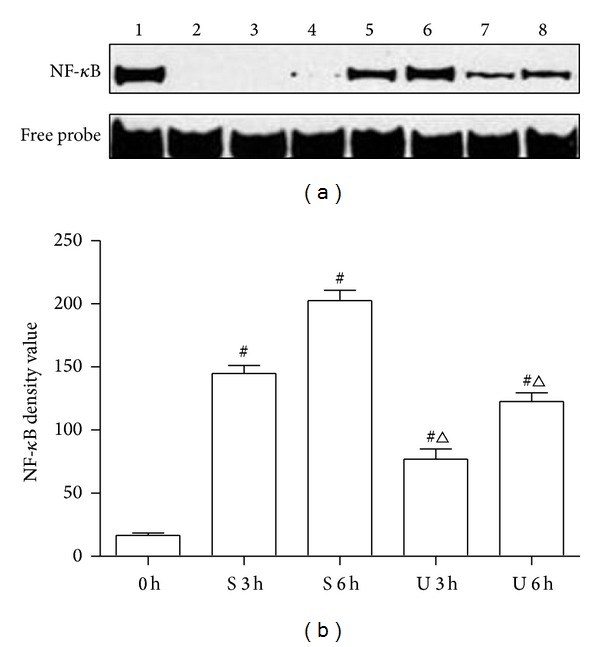
Effects of UTI on inhibition of NF-*κ*B activity. (a) Shifted band of NF-*κ*B DNA complexes. Lane 1: positive control, lane 2: negative control, lane 3: cold competition assay, lane 4: C group, lane 5: S group at 3 h, lane 6: S group at 6 h, lane 7: U group at 3 h, and lane 8: U group at 6 h. (b) Amount of NF-*κ*B DNA complexes. 0 h = C group; S 3 h = S group at 3 h; S 6 h = S group at 6 h; U 3 h = U group at 3 h; U 6 h = U group at 6 h. ^#^ *P* < 0.05 versus C group and ^△^ *P* < 0.05 versus S group at matched time.

**Figure 4:**
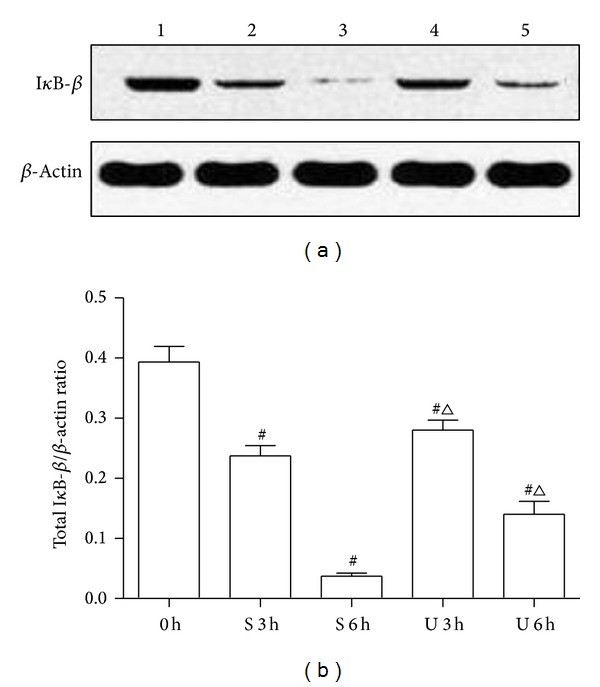
Effects of UTI on inhibition of I*κ*B-*β*. (a) Lane 1: C group, lane 2: S group at 3 h, lane 3: S group at 6 h, lane 4: U group at 3 h, and lane 5: U group at 6 h. (b) Amount of I*κ*B-*β*. 0 h = C group; S 3 h = S group at 3 h; S 6 h = S group at 6 h; U 3 h = U group at 3 h; U 6 h = U group at 6 h. ^#^ *P* < 0.05 versus C group and ^△^ *P* < 0.05 versus S group at matched time.

The translocation of the transcription factor NF-*κ*B to the nucleus in intestine was determined by EMSA. As shown in Figure 3, there was a very low NF-*κ*B binding activity in the rats of C group, which showed almost undetectable band intensity in EMSA autoradiograph. SI-OAI induced significant increase of NF-*κ*B translocation in S and U groups compared with those in C group (*P* < 0.05). UTI treatment obviously inhibited the NF-*κ*B translocation in intestine compared with S group (*P* < 0.05). We measured protein levels of I*κ*B-*β* using Western blot analysis since it had been well demonstrated that activation of NF-*κ*B correlates with rapid proteolytic degradation of I*κ*B. As shown in Figure 4, I*κ*B-*β* levels were decreased in rats after SI-OAI compared with C group and this effect was partially blocked by UTI (*P* < 0.05).

### 3.5. Histomorphometric Studies

**Figure 5:**
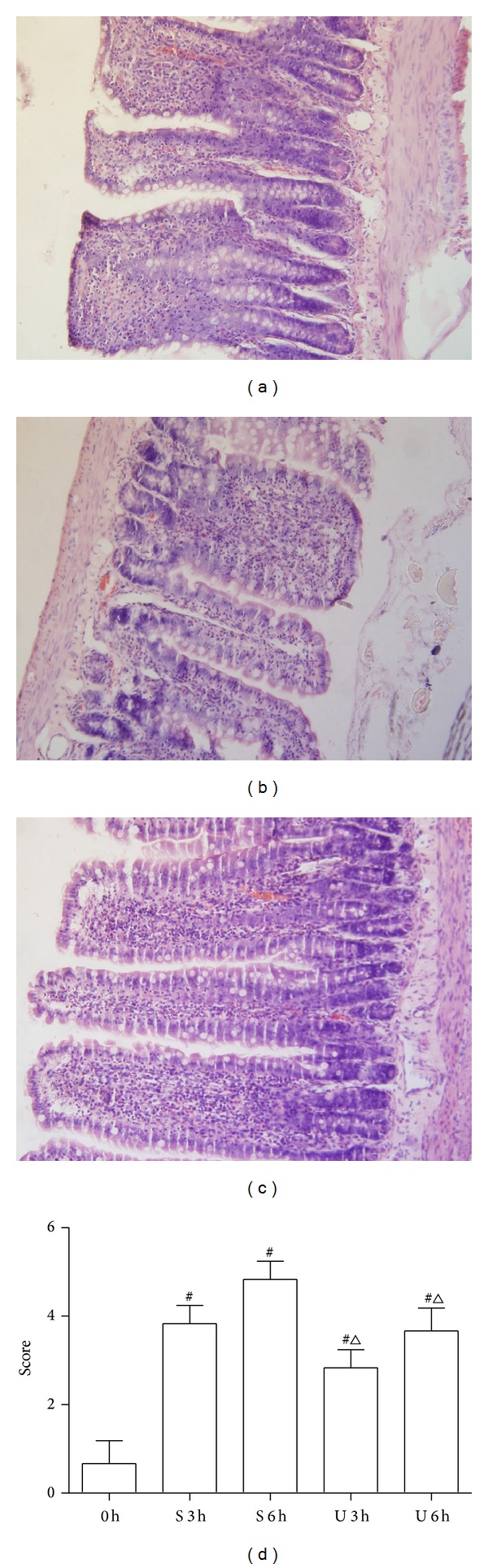
Effects of UTI on the pathological alteration of rat intestinal (light microscope, H&E stain, and original magnification ×100). (a) C group; (b) S group at 6 h; (c) U group at 6 h; (d) Chiu's grade scores: 0 h = C group; S 3 h = S group at 3 h; S 6 h = S group at 6 h; U 3 h = U group at 3 h; U 6 h = U group at 6 h. ^#^ *P* < 0.05 versus C group and ^△^ *P* < 0.05 versus S group at matched time.

Histopathologic findings showed the morphology of intestinal mucosa was approximately normal in control groups. Seawater immersion caused acute intestinal mucosa injury and inflammation which were represented by massive destruction of villi and inflammatory cell infiltration into the lamina propria in rats of S and U groups. Histological injury scores in rats of U group were significantly lower compared to S group (Figures 5(a)–5(c)). In addition, the total pathological scores were significantly decreased by UTI treatment (Figure 5(d)).

## 4. Discussion

Abdominal trauma is one of the most common acute injuries in modern battles. The rate of abdominal injury was 7.8% in United States Armed Forces during Operation Iraqi Freedom [22]. SI-OAI is far more complicated than ordinary abdominal injury. It could lead to severe disorders in metabolism and hemodynamics owning to the characteristics of the higher osmotic pressure and the lower temperature in seawater [7, 23]. Besides, IMI in the pathogenesis of SI-OAI has gained extensive attention in recent years [5, 6]. The intestinal barrier is the largest and most important barrier against the invasion of intestinal bacteria and endotoxin [1, 24]. Disruption of intestinal barrier during SI-OAI occurs with increased intestinal permeability, bacteria translocation, and high plasma level of endotoxin, starting the SIRS and finally leading to multiple organ failure with a high mortality rate [9, 25]. Although IMI in onset and progression of SI-OAI has been well understood, up to now, there is no effective treatment to significantly prevent it. In the current study, we explore the therapeutic effect of UTI on SI-OAI induced IMI.

UTI, an endogenous protease inhibitor and a neutral protease activator, has varieties of therapeutic mechanisms: (1) suppression of the elastase release from neutrophils by stabilizing lysosomal membranes and suppressing the release of lysosomal enzymes; (2) anti-inflammation by inhibiting the production of cytokines and adhesion molecules; (3) antioxidation [26–28]. In the present study, we found that UTI treatment significantly inhibited the expression of NF-*κ*B, decreased the level of I-*κ*B protein, and suppressed the release of related inflammatory mediators and the generation of ROS in rat models of SI-OAI. Moreover, it significantly ameliorated the histopathological damage of gut mucosal injury induced by seawater immersion, highlighting the protective effect of UTI in the treatment of IMI induced by SI-OAI.

Additionally, we also investigated the possible mechanism of UTI in protecting against IMI induced by SI-OAI. Accumulating evidences supported that the imbalance of proinflammatory cytokines and anti-inflammatory cytokines during SI-OAI is the main cause of IMI [8, 9]. Compared with other cytokines, the classic proinflammatory cytokine TNF-*α* and IL-1 play important roles in the early period of inflammatory response [29]. TNF-*α* and IL-1 can be immediately released by macrophages during the early steps of inflammation, stimulating endothelial cells and macrophages to release other inflammatory cytokines. They also play important roles in inducting expression of adhesion molecules on both polymorphonuclear neutrophils and endothelial cells, causing increased leukocyte/endothelial adherence and activation of leukocytes, thus finally resulting in local tissue damage [30, 31]. In the pathogenesis of intestinal ischemia-reperfusion injury, TNF-*α* and IL-1 play important roles in the progress of intestinal mucosa injury [32]. In this study, very high levels of TNF-*α* and IL-1 were observed in rat models of SI-OAI, and these changes in cytokine levels were significantly ameliorated by UTI treatment, suggesting that UTI may have anti-inflammatory effect in SI-OAI.

Oxidative stress, which plays a “trigger” role in the pathophysiological process of IMI, is characterized by an imbalance between ROS and the antioxidative defense system [33, 34]. It has been shown in many studies that SI-OAI is associated with lipid peroxidation and a damage of antioxidant defense system [10]. To study the effect of UTI on oxidative stress, we tested the levels of MDA, SOD, and ROS in intestine. MDA is considered to be a good indicator of oxidative injury and an end product of lipid peroxidation. SOD is an endogenous superoxide anion radical scavenger, which has been widely used to evaluate the body's ability of eliminating free radicals [16, 35]. Our studies showed that UTI treatment significantly decreased the levels of MDA and ROS and increased the level of SOD in rat models of SI-OAI, suggesting that UTI protects against oxidative damage induced by SI-OAI, and this effect possibly involves the inhibition of lipid peroxidation and the elimination of free radicals.

NF-*κ*B proteins are a family of transcription factors that play essential roles in cell adhesion and immune and proinflammatory responses. The mammalian NF-*κ*B family consists of five members, including RelA (also named p65), RelB, c-Rel, NF-*κ*B1 p50, and NF-*κ*B2 p52, all of which form homo- or heterodimers in the cytoplasm. NF-*κ*B proteins are normally bound to inhibitory molecules of the I*κ*B family of proteins as an inactive complex. Inflammatory cytokines, such as TNF-*α* and IL-6, can activate the canonical NF-*κ*B pathway, start the phosphorylation and degradation of the I*κ*B protein, particularly I*κ*B*α*, and cause the onset of NF-*κ*B translocation to the nucleus and target gene transcription [36–38]. In addition, NF-*κ*B also can be directly activated by oxidant stimulation through the phosphorylation and degradation of I*κ*B in the cytoplasmic NF-*κ*B-I*κ*B complex [39, 40]. Proinflammatory cytokines and ROS, promote each other in the activation of NF-*κ*B and generate a vicious circle in SI-OAI. In the current study, data showed an obvious rise in generation of ROS and inflammatory cytokines; nuclear translocation of NF-*κ*B after SI-OAI supported the notion that the generation of ROS and inflammatory cytokines may be responsible for activation of NF-*κ*B after SI-OAI. Our data showed that administration of UTI significantly suppressed the excessive production of NF-*κ*B caused by seawater immersion. In the previous studies on UTI and NF-*κ*B, Yamaguchi et al. [41] reported that UTI had no effect on NF-*κ*B activation since it did not prevent degradation of I*κ*B-*α* in MCF7 human breast carcinoma cells. On the contrary, recently, studies had demonstrated that UTI could reduce NF-*κ*B activation, at least in part, by inhibiting the expression of I*κ*B [42, 43]. Here, we demonstrated the level of I*κ*B-*β* was degraded in intestinal mucosa of SI-OAI rat and UTI could protect against it. These data indicated that UTI could inhibit I*κ*B-*β* degradation, while indirectly reducing NF-*κ*B activation. However, the mechanisms of inhibitory effects of NF-*κ*B activity were various; both downregulation of inflammatory cytokines expression and suppression of the generation of ROS could weaken the positive feedback mechanism of NF-*κ*B, which indicated that the inhibition effect on NF-*κ*B activation by UTI may have other pathways [44, 45]. Further studies are needed to get a further insight into the molecular mechanism of the effect of UTI on NF-*κ*B activity.

In summary, SI-OAI can trigger a cascade of responses, including the activation of NF-*κ*B, the production of inflammatory cytokines, and oxidative stress. UTI mainly achieved the goal of protecting intestinal mucosa by suppressing oxidative stress, reducing the NF-*κ*B activation, and subsequently inhibiting the expression of inflammatory cytokines. These results suggested UTI had a remarkable protective effect on SI-OAI induced IMI.
